# Controlled intramyocardial release of engineered chemokines by biodegradable hydrogels as a treatment approach of myocardial infarction

**DOI:** 10.1111/jcmm.12225

**Published:** 2014-02-06

**Authors:** Delia Projahn, Sakine Simsekyilmaz, Smriti Singh, Isabella Kanzler, Birgit K Kramp, Marcella Langer, Alexandrina Burlacu, Jürgen Bernhagen, Doris Klee, Alma Zernecke, Tilman M Hackeng, Jürgen Groll, Christian Weber, Elisa A Liehn, Rory R Koenen

**Affiliations:** a Institute for Molecular Cardiovascular Research (IMCAR), Medical Faculty, RWTH Aachen UniversityAachen, Germany; b Institute for Cardiovascular Prevention (IPEK), University Hospital of the LMU MunichMunich, Germany; c Interactive Material Research - DWI an der RWTH Aachen e.V and Institute for Technical and Macromolecular ChemistryAachen, Germany; d Institute of Cellular Biology and Pathology “Nicolae Simionescu” of the Romanian AcademyBucharest, Romania; e Institute of Biochemistry and Molecular Cell BiologyAachen, Germany; f Department of Vascular Surgery, Klinikum rechts der Isar, Technical University MunichMunich, Germany; g Department and Chair of Functional Materials in Medicine and Dentistry, University of WürzburgWürzburg, Germany; h Munich Heart AllianceMunich, Germany; i Department of Biochemistry, Cardiovascular Research Institute Maastricht (CARIM), Maastricht UniversityMaastricht, The Netherlands

**Keywords:** heart failure, chemokines, therapy, cardiovascular pharmacology, remodelling

## Abstract

Myocardial infarction (MI) induces a complex inflammatory immune response, followed by the remodelling of the heart muscle and scar formation. The rapid regeneration of the blood vessel network system by the attraction of hematopoietic stem cells is beneficial for heart function. Despite the important role of chemokines in these processes, their use in clinical practice has so far been limited by their limited availability over a long time-span *in vivo*. Here, a method is presented to increase physiological availability of chemokines at the site of injury over a defined time-span and simultaneously control their release using biodegradable hydrogels. Two different biodegradable hydrogels were implemented, a fast degradable hydrogel (FDH) for delivering Met-CCL5 over 24 hrs and a slow degradable hydrogel (SDH) for a gradual release of protease-resistant CXCL12 (S4V) over 4 weeks. We demonstrate that the time-controlled release using Met-CCL5-FDH and CXCL12 (S4V)-SDH suppressed initial neutrophil infiltration, promoted neovascularization and reduced apoptosis in the infarcted myocardium. Thus, we were able to significantly preserve the cardiac function after MI. This study demonstrates that time-controlled, biopolymer-mediated delivery of chemokines represents a novel and feasible strategy to support the endogenous reparatory mechanisms after MI and may compliment cell-based therapies.

## Introduction

Myocardial infarction (MI), following the occlusion of an atherosclerotic coronary artery, causes death of the cardiomyocytes and initiation of an inflammatory reaction, activation of the nuclear factor (NF)-κB and toll-like receptor (TLR)-mediated pathways, up-regulation of chemokines, cytokines as well as adhesion molecules in endothelial cells and leucocytes [1]. All these processes lead to the infiltration of polymorphonuclear cells, monocytes and lymphocytes into the infarcted area and are of crucial importance in the proper healing and scar formation. Neutrophil- and macrophage-derived inflammatory signals initiate the phagocytosis of dead cells and matrix degradation products. During the proliferation phase, the expression of inflammatory mediators is inhibited; the fibroblasts and endothelial cells infiltrate into the infarcted area and promote collagen deposition and angiogenesis. The cardiac repair, or so-called ‘remodeling’, of the heart muscle is in fact the replacement of the adjacent myocardium with connective tissue and consecutive dilation of the ventricles [1,2].

Despite the extensive progress in the past decades, current clinical therapies are not always efficient in reducing myocardial necrosis and optimizing cardiac repair following infarction. New strategies have been proposed such as progenitor cell therapy, which was considered as a promising therapeutic approach to provide an alternative way to regenerate the heart structure after MI. Pioneering studies in animals have shown that transplantation of adult stem cells after acute MI improves neovascularization and reduces fibrosis, preserving the left ventricular (LV) heart function [3,4]. Unfortunately, these promising results were less pronounced in clinical studies in patients [5], probably because of the difficult translation of the experimental findings from the animal to the human system. New data suggest that an additional mechanism of improving the heart function by cell-based therapies can be ascribed to inflammation and its consecutive effects [6]. These findings are of significance, suggesting that we can potentially improve the outcome after cell therapy by selectively controlling and manipulating not only the trafficking and behaviour of the transplanted cells but also the inflammatory reaction through the modulation of chemokine responses [2].

Chemokines are small signalling proteins that regulate the trafficking of a large variety of cell types [7]. They can both prevent apoptosis of cardiomyocytes and promote angiogenesis in the infarcted area [2]. The inhibition of neutrophil infiltration by blocking CCR1 or by Met-CCL5 (Met-RANTES) treatment leads to improved LV function and reduction in infarct extension [8,9], possibly by decreasing myeloperoxidase expression and preventing further increase in the resulting tissue damage [10]. CXCL12 (Stromal Cell-Derived Factor 1/SDF-1) and its protease-resistant mutants are well-established agents for recruiting hematopoietic stem cells from the circulating blood, resulting in a significant increase in vessel formation, vascularization in the scar tissue and a consecutive improvement of LV function after MI [11–13].

In this study, we propose a novel combined approach using biodegradable synthetic hydrogels [14,15] to achieve spatially and temporally controlled delivery of Met-CCL5 and recombinant protease-resistant CXCL12 (S4V), two established agents that inhibit neutrophil infiltration and improve hematopoietic stem cell recruitment to sustain neovascularization respectively. A very rapid biodegradable hydrogel assured a faster release and action of Met-CCL5 to block neutrophil infiltration during the first hours. A second slower biodegradable hydrogel served to ensure a longer term release and action of CXCL12 (S4V) to attract hematopoietic stem cells over several weeks. Thus, this presents a novel approach for the prevention of tissue damage after MI.

## Materials and methods

### Construction of CXCL12 and Met-CCL5 expression plasmids

The DNA sequence of CXCL12 was cloned from mouse peripheral blood mononuclear cells (PBMC) cDNA into the pBluescript II KS (Stratagene, La Jolla, CA, USA) and mutations were applied in the N-terminus using QuikChange® II site–directed mutagenesis kit (Stratagene) according to manufacturer*s instructions. This resulted in CXCL12 (S4V) variant and inactive control CXCL12 (S2G4V) resistant to MMP-2 and DPPIV enzymatic cleavage as reported previously by Segers *et al*. [13]. The cDNAs were subcloned into pET32a (Merck, Darmstadt, Germany) for expression as thioredoxin fusion proteins in *Escherichia coli* (see below). For the same purpose, Met-CCL5 was ordered as a codon-optimized synthetic gene cloned in pET26+ (Merck) from Genscript (Piscataway, NJ, USA). Detailed procedures can be found in the online supplementary information.

### Expression and purification of recombinant chemokines

The recombinant chemokines were expressed in and purified from *E. coli* Rosetta DE3 (Merck) as described previously [16–18]. After purification, the chemokines were dialysed in >100 volumes of 0.01% trifluoroacetic acid and lyophilized for long-term storage.

### Isolation of early-outgrowth cells and neutrophils from peripheral blood

Angiogenic early-outgrowth cells (EOC) were isolated according to established protocols [19–21] from citrate/dextran anticoagulated peripheral blood buffy coats of healthy volunteers. Peripheral blood mononuclear cells were separated by density gradient centrifugation with Biocoll (Merck). The PBMC were washed twice with PBS, resuspended in endothelial cell growth medium MV2 and plated on fibronectin-coated (10 μg/ml) 6-well plates (10^7^ cells per well). At day 4, the medium was changed and the adherent cells were detached with Accutase for 5 min. at 37°C, counted and subjected for activity assay at day 5–7. Neutrophils were isolated from blood collected in the presence of EDTA (1.6 mg EDTA/ml blood) according to established protocols using Polymorphprep™ (Axis-Shield, Oslo, Norway). Experiments with human material were approved by the local ethics board and all individuals gave informed consent.

### Chemotaxis experiments

Activities of purified CXCL12 (S4V), CXCL12 (S2G4V) and Met-CCL5 were assayed by migration of EOC (for CXCL12 variants) or neutrophils (for Met-CCL5) and compared with commercially available chemokines. CXCL8 is an established neutrophil attractant and used as positive control for neutrophil adhesion. Cells (500,000 cells/ml) were added to the upper well of 6.5 mm transwell™ inserts with 8.0 μm pore polycarbonate membranes (Costar, Tewksbury, MA, USA), and chemokines (200 ng/ml) were added to the lower wells. Cells were counted by flow cytometry (FACS Canto II; BD Biosciences, San Jose, CA, USA) in the lower well after 1 hr (neutrophils) or 3 hrs (EOC). All experiments were performed in triplicate.

### Cell adhesion assays under flow conditions

Flow-resistant adhesion on endothelial cells in response to recombinant chemokines was assessed in customized flow chambers as described by Postea *et al*. [18]. Briefly, Jurkat T lymphoma cells or neutrophils were labelled by the addition of calcein (1 μM; Life Technologies, Carlsbad, CA, USA). Chemokines were added (200 ng/ml) and incubated for 5 min. at 37°C. Human umbilical vein endothelial cells (HUVEC), cultured in 35-mm dishes, were activated with tumour necrosis factor-α (10 ng/ml) for 4 hrs and assembled into flow chambers. Cells (500,000 cells/ml) were perfused for 3 min. at a wall shear stress of 1.5 dynes/cm^2^ in Hank*s buffer containing HEPES (10 mmol/l), CaCl_2_ and MgCl_2_ (1 mmol/l each) and 0.5% human albumin (Baxter, Deerfield, IL, USA) subsequently followed by video microscopic quantification of adherent fluorescent cells. Adherent cell were manually counted in at least six fields and expressed as cells/mm^2^.

### Synthesis of biodegradable hydrogels

The synthesis of hydrogels from thiol-functionalized biodegradable sP(EO-*stat*-PO) pre-polymer was performed as described previously [22]. Simple oxidation of thiolated pre-polymer by H_2_O_2_ resulted into fast degradable hydrogel (FDH) with disulphide bonds, while the crosslinking of thiols by Michael addition with added PEG-diacrylate resulted in a slowly degrading hydrogel (SDH; Fig. S1) with thioether bonds.

### Release of CXCL12 (S4V) and Met-CCL5 from biodegradable hydrogels

Slowly degrading and fast degrading hydrogels (15 μl) containing 3 μmol/l CXCL12 (S4V) or 0.5 μmol/l Met-CCL5, respectively, were incubated with 250 μl PBS without or with 5 mmol/l reduced glutathione (Sigma-Aldrich, St. Louis, MO, USA) and incubated over 4 weeks (SDH) or 24 hrs (FDH). At suitable time-points, 250 μl PBS containing 5 mmol/l reduced glutathione was replaced and the supernatant was analysed by DuoSet ELISA kits (R&D Systems, Minneapolis, MN, USA) for mouse CXCL12/SDF-1 or human CCL5/RANTES according to the manufacturer*s instructions.

### Biocompatibility of CXCL12 (S4V), Met-CCL5 and biodegradable polymers

Human umbilical vein endothelial cells were seeded in collagen-G–coated 96-well plates with Endothelial Cell Growth Medium (PromoCell, Heidelberg, Germany) and cells were allowed to attach prior to treatment with 5 μg/ml CCL5, 30 μg/ml CXCL12 (S4V) and 0.45 g/ml biodegradable hydrogels. Proliferation between 0 and 72 hrs was determined using BrdU Proliferation Assay (Novagen/Merck Bioscience, Darmstadt, Germany) and cytotoxicity was detected using the CellTiter-Blue® cell viability assay (Promega, Mannheim, Germany) at time-points ranging from 0 to 24 hrs. All procedures were performed according to the manufacturer*s instructions. The data were expressed as the relative signals (RFU) after subtraction of the signals of the appropriate buffer or medium controls.

### Mouse model of myocardial infarction and injection of biodegradable hydrogels containing chemokines

Eight-week-old male littermate C57BL/6 mice (25–26 g, *n* = 6–9 per group) were randomly subjected to coronary occlusion as described earlier [9,23]. Only mice dying during the operation as a result of the surgery complications were excluded from the statistical measurements. Briefly, mice were intubated under general anaesthesia (using ketamine and xylazine) and positive pressure ventilation was maintained using a rodent respirator. Hearts were exposed by left thoracotomy and MI is induced by suture occlusion of the left anterior descending artery over a silicone tube. Biodegradable hydrogels SDH and FDH (15 μl) were mixed with crosslinking agent and loaded with buffer or 0.5 μg Met-CCL5 and/or 3 μg CXCL12 (S4V) and subsequently injected separately in a standardized manner, using a 36-gauge needle into two directly adjacent sites of the mouse myocardium at the border of the infarct area directly after inducing MI. Control mice (*n* = 6) received MI with injected PBS in equal volumes. Hydrogels are not thermo-responsive; the components are mixed shortly before transplantation and will gel on site immediately after injection. Fast degradable hydrogel degrades in 24 hrs *in vivo*, whereas SDH needs 4 weeks for complete degradation. The gels do not evoke immune reactions and some studies describe anti-inflammatory properties of the gels [14,15]. Therefore, control groups with or without gels were included. The muscle layer and skin incision were closed with a silk suture, after polymerization was complete. The animals were treated with a single dose of buprenorphine (0.1 mg/ml) and kept under standard conditions 1 day or 4 weeks after MI until further investigation. Heart function of the mice was evaluated by echocardiography 1 day before, and 4 weeks after MI. All animal experiments and study protocols were approved by local authorities, complying with Romanian animal protection laws.

### Quantitative immunohistochemistry and immunofluorescence

The infarcted area was determined in serial sections (5 μm) of the infarcted myocardium (10 sections per mouse) after staining with Gomori*s 1-step trichrome staining using Diskus software (Hilgers, Königswinter, Germany) and expressed as percentage of LV area. The number of neutrophils (naphthol-AS-D-chloroacetate esterase and anti-MPO, #RB-373-A; Thermo Scientific, Waltham, MA, USA) was determined 1 day after MI, whereas the endothelial cells (CD31, #M20; Santa Cruz, Santa Cruz, CA, USA) was determined in formalin-fixed serial sections (three per mouse, 200 μm apart) of the infarcted myocardium 4 weeks after MI. Positive-stained cells were numbered in six different fields from infarcted area per section and expressed as cells/mm^2^. Blood vessels positive for CD31 were quantified and expressed as CD31/mm^2^. Apoptotic (TUNEL, TMRred; Roche, Mannheim, Germany) and proliferating (Ki67, clone TEC-3; DAKO, Hamburg, Germany) cells were stained, counted and expressed as per cent from total cells (DAPI staining). Total Akt (Akt1/2 (N-19), sc-1619; Santa Cruz) and phosphorylated-Akt (phospho-Akt (Ser) (193H12), #4058S; Cell Signaling Technologies, Beverly, MA, USA) were stained using Dylight 488–conjugated secondary antibodies (Thermo Scientific).

### Echocardiographic measurements

Two-dimensional and M-mode echocardiographic measurements (Vevo 770; Visual Sonics, Toronto, ON, Canada) were performed before and 4 weeks after induction of MI. Mice are anesthetized with 1.5% isoflurane (2-chloro-2-(difluoromethoxy)-1,1,1-trifluoro-ethane) *via* mask and placed in supine position on a warming pad. The ejection fraction (EF) was recorded and analysed in long axis and orthogonally in the short axis; the average of both results was used for further analysis [23]. LV dimensions in systole and diastole were also measured using M-Mode in the short axis (Table 1A and B).

**Table 1:** Echocardiographic baseline measurements (*n* = 6-9 mice; A). Echocardiographic parameters 4 weeks after MI (B)

	Control	FDH SDH	Met-CCL5-FDH	CXCL12-SDH	Met-CCL5-FDH CXCL12-SDH
(A)					
EF (%)	59.2 ± 3.08	57.3 ± 3.33	56.7 ± 1.02	53.8 ± 0.82	55.6 ± 1.13
Diastolic LVD (mm)	3.97 ± 0.25	3.73 ± 0.19	3.34 ± 0.16	3.75 ± 0.22	3.85 ± 0.12
Systolic LVD (mm)	2.90 ± 0.28	2.91 ± 0.21	2.57 ± 0.10	2.92 ± 0.26	2.92 ± 0.11
Heart rate (BMP)	410 ± 29.8	380 ± 23.3	403 ± 24.4	379 ± 10.9	371 ± 7.73
Heart weight (mg)	99 ± 4.60	106 ± 10.8	95 ± 6.01	98 ± 14.7	107 ± 6.19
(B)					
EF (%)	35.8 ± 1.85***	31.6 ± 1.30	41.3 ± 2.31**	40.5 ± 1.83***	50.1 ± 1.59*
Diastolic LVD (mm)	5.66 ± 0.28	6.48 ± 0.15	6.35 ± 0.37	5.87 ± 0.62	6.56 ± 0.10
Systolic LVD (mm)	4.57 ± 0.49	5.52 ± 0.22	4.88 ± 0.46	4.53 ± 0.57	4.74 ± 0.45
Heart rate (BMP)	442 ± 11.4	426 ± 38.2	501 ± 36.7	502 ± 34.4	414 ± 34.9
Heart weight (mg)	107 ± 6.58	119 ± 7.93	100 ± 8.77	104 ± 6.70	103 ± 4.86

*P < 0.01, 0.001 *versus* FDH SDH respectively; *n* = 6–9; **, ***P < 0.01, 0.001 *versus* Met-CCL5-FDH+CXCL12 (S4V)-SDH, respectively; *n* = 6–9 mice; anova.

EF: ejection fraction; LVD: left ventricular diameter; FDH: fast degradable hydrogel; SDH: slow degradable hydrogel; MIL: myocardial infarction.

### Statistical analysis

Data were represented as mean value ± SE. Data analysis was performed with Prism 4 software (Graph Pad Software, San Diego, CA, USA) using one-way parametric anova followed by Newman-Keuls post hoc testing or Kruskall–Wallis non-parametric testing with Dunn*s post hoc comparison, where appropriate. Differences with *P* < 0.05 were considered significant.

## Results

### Generation and functional analysis of chemokines and biodegradable gels

**Fig. 1:**
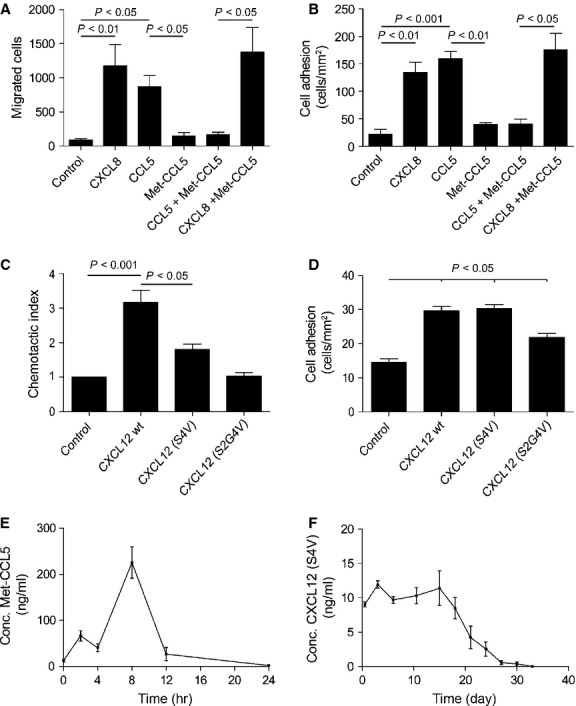
*In vitro* studies of recombinant chemokines. (**A**) Chemotaxis assay of neutrophils towards Met-CCL5, CCL5 *wild-type* and commercially available CXCL8 *wild-type*. (**B**) Adhesion assay under flow on activated human umbilical vein endothelial cells (HUVEC) using Met-CCL5-, CCL5 *wild-type* and CXCL8-stimulated neutrophils. (**C**) Chemotactic index of angiogenic early-outgrowth cells towards CXCL12 (S4V and S2G4V) and commercially available CXCL12 *wild-type*. (**D**) Adhesion assay using CXCL12 (S4V and S2G4V)-stimulated Jurkat cells on activated HUVEC under flow conditions in comparison with the commercially available CXCL12 *wild-type*. (**E**) Release of the Met-CCL5 chemokine from the fast degradable hydrogel over 24 hrs and (**F**) of the CXCL12 (S4V) chemokine from the slow degradable hydrogel over 33 days. Depicted P values are based on parametric (**A**, **B**, **D**) or non-parametric (**C**) anova (*n* = 6–9).

Recombinant Met-CCL5, CXCL12 (S4V) and CXCL12 (S2G4V) were expressed in and purified from *E. coli*. SDS-page, western blot and MALDI-TOF analysis confirmed the purities (>95%), identity and the expected masses of 7.98, 8.07 and 8.03 kD for Met-CCL5, CXCL12 (S4V) and CXCL12 (S2G4V; Figs S2 and 3). The activities of the recombinant chemokines were assessed using cell-recruitment assays with the designated target cell types. Isolated neutrophils stimulated with CXCL8 and/or CCL5 showed considerable chemotaxis and flow-resistant adhesion to HUVEC (Fig. 1A and B). Met-CCL5 specifically blocked the action of CCL5, but not of CXCL8 (an established neutrophil attractant). The recombinant CXCL12 (S4V) induced the migration of angiogenic EOC to a slightly lower extent than CXCL12 *wild-type*, whereas the activity of CXCL12 (S2G4V) was comparable with the negative control (Fig. 1C). However, the adhesion of CXCL12 (S4V)-stimulated Jurkat cells onto HUVEC under flow conditions was comparable with CXCL12 *wild-type*. CXCL12 (S2G4V) showed significantly less adhesion compared with the CXCL12 (S4V) and CXCL12 *wild-type* (Fig. 1D). Neither treatment with Met-CCL5 nor with CXCL12 (S4V) impaired the proliferation and viability of cultured HUVEC cells (Fig. S4A–D).

To control the local release of the protease-resistant CXCL12 and Met-CCL5, two different biodegradable hydrogels were utilized. Six arm, star-shaped polymers with copolymerized ethylene oxide and propylene oxide in the ratio of 4 to 1, termed sP(EO-*stat*-PO), were chosen as precursors for both types of hydrogel. Of six arms of the polymer, three were functionalized with thiol groups, which were crosslinked in two ways, resulting in FDH or SDH respectively (Fig. S1). The FDH is used for the formulation with Met-CCL5 for a quick release to inhibit neutrophil infiltration within the first hour after MI. This fast release of Met-CCL5 from FDH was confirmed by incubation in PBS and 5 mmol/l glutathione, which led to complete degradation of the gel after 24 hrs (Fig. 1E) as a result of disulphide bond cleavage. The SDH was conceived for formulation with the protease-resistant CXCL12 (S4V) for a gradual release to recruit hematopoietic stem cells from the circulating blood during a time period of 4 weeks after MI. To confirm the release rate, the CXCL12 (S4V)-SDH was incubated for 33 days in PBS to detect the local release over time. After 33 days, the SDH was entirely degraded because of the hydrolytic cleavage of ester bonds in the backbone and release of chemokine was no longer detected (Fig. 1F). Proliferation and viability of HUVEC were not affected by culturing in the presence of the thiolated polymer (Fig. 4E and F).

### Improvement of cardiac function after MI in mice by combined treatment with the protease-resistant CXCL12 and Met-CCL5

To investigate a possible beneficial role of a blocked short-term neutrophil influx after cardiac ischaemia combined with a longer term recruitment of hematopoietic cells, the chemokine-loaded hydrogels were applied in an experimental model of MI. During hydrogel treatment, the levels of CXCL12 (S4V) in mouse sera after 1 day and 4 weeks were not increased (Fig. 5A and B), because of the very slow release rate from the hydrogel and the local application. Human Met-CCL5 levels were increased after 1 day of treatment with Met-CCL5–containing FDH (Fig. S5C), because of the quick release of human Met-CCL5 from these hydrogels. As expected, the human Met-CCL5 was no longer detected in the mouse sera after 4 weeks (Fig. S5D). The concentrations of mouse CCL5 in sera were slightly increased after administration of any hydrogel variant (Fig. S5E and F).

**Fig. 2:**
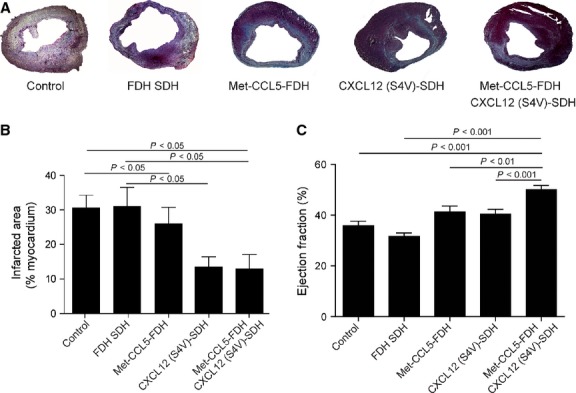
Assessment of cardiac tissue damage and function after experimental myocardial infarction (MI). Representative Gomori-stained sections of the hearts (**A**) and histomorphometric quantifications (**B**) of Control, fast degradable hydrogel slow degradable hydrogel (FDH SDH), Met-CCL5-FDH, CXCL12 (S4V)-SDH and Met-CCL5-FDH CXCL12 (S4V)-SDH-treated mice (*n* = 6–9 per group), 4 weeks after MI. Echocardiographic measurements of ejection fraction (EF; **C**) of Control, FDH SDH, Met-CCL5-FDH, CXCL12 (S4V)-FDH and Met-CCL5-FDH+CXCL12 (S4V)-FDH-treated mice (*n* = 6–9 per group), 4 weeks after MI. Depicted P values are based on anova.

After 4 weeks, the infarcted area was notably reduced in the CXCL12 (S4V)-SDH (13.4 ± 2.85%) and in the combined Met-CCL5-FDH+CXCL12 (S4V)-SDH group (12.8 ± 4.18%), compared with the groups that received hydrogel only (30.7 ± 5.51%) and Met-CCL5-FDH (25.7 ± 4.73%; Fig. 2A and B). Administration of the hydrogel itself did not affect infarct size, as the infarcted area of the control group was similar (30.3 ± 3.72%; Fig. 2A and B).

The EF was increased in the group with Met-CCL5-FDH and CXCL12 (S4V)-SDH in comparison with the hydrogel and control group, but the combined treatment with Met-CCL5-FDH + CXCL12 (S4V)-SDH showed the highest preservation of the heart function comparing with all other groups (Fig. 2C, Table 1). No changes in baseline parameters (before MI, Table 1A), as well as in heart weight and systolic or diastolic LV diameters before (Table 1A) and 4 weeks after MI, were observed between the treated groups (Table 1B). However, evaluation of the functional parameters of the heart revealed a significant improvement of cardiac function in the Met-CCL5-FDH + CXCL12 (S4V)-SDH group that received a combined treatment (Table 1B and Fig. 2C).

**Fig. 3:**
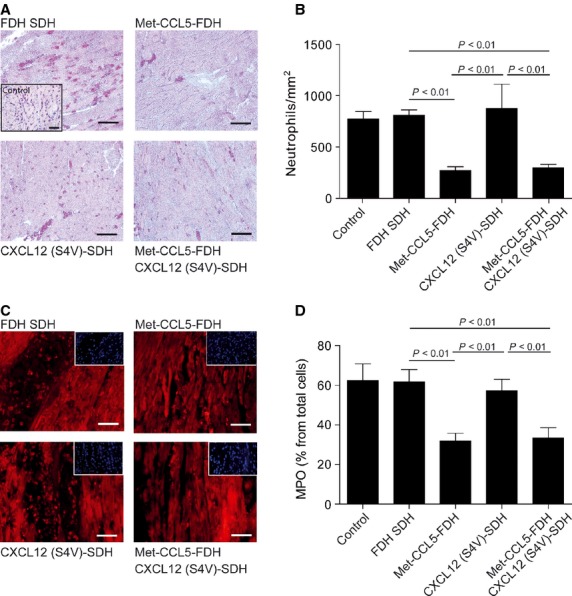
Assessment of neutrophil infiltration after experimental myocardial infarction (MI). Neutrophil infiltration in the myocardium 1 day after MI by esterase-staining (**A**) and quantification (**B**) and MPO-staining (**C**) and -quantification (**D**) of fast degradable hydrogel slow degradable hydrogel (FDH SDH), Met-CCL5-FDH, CXCL12 (S4V)-FDH and Met-CCL5-FDH+CXCL12 (S4V)-FDH-treated mice (*n* = 6–9 per group). Insets in (**A**) and (**B**) show negative control staining; scale bars: 50 μm. Depicted P values are based on non-parametric anova (n ≥ 6).

**Fig. 4:**
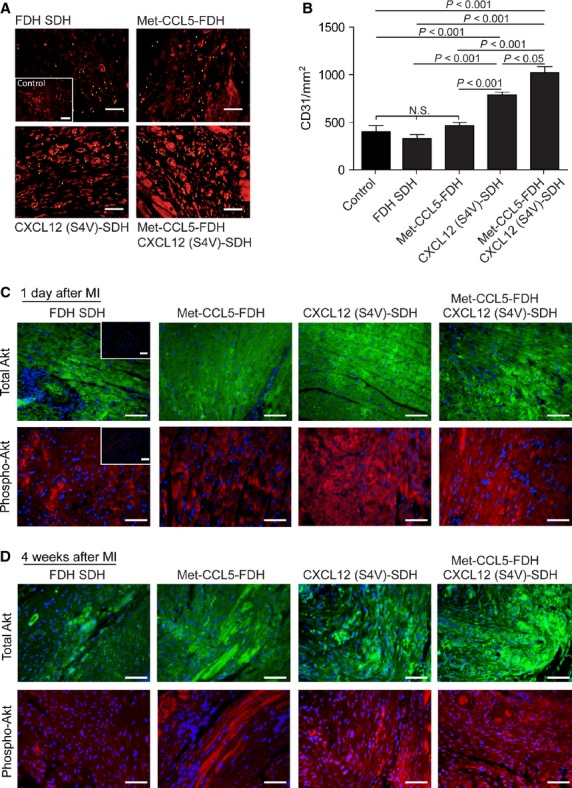
Assessment of angiogenesis after experimental myocardial infarction (MI). Neovascularization as quantified by representative CD31 staining 4 weeks after MI (**A**) and quantification (**B**) of fast degradable hydrogel slow degradable hydrogel (FDH SDH), Met-CCL5-FDH, CXCL12 (S4V)-FDH and Met-CCL5-FDH+CXCL12 (S4V)-FDH-treated mice (*n* = 6–9 per group). Examples of stained capillaries are marked with triangles; scale bars: 50 μm. Depicted P values are based on parametric anova (*n* ≥ 6). Immunohistological staining of total Akt (green) and phosphorylated (phospho-) Akt (red), 1 day (**C**) and 4 weeks (**D**) after MI. Insets represent negative controls; scale bars: 50 μm.

The same applied for neutrophil recruitment (Fig. 3) and neovascularization (Fig. 4). The immediate infiltration of MI-induced neutrophils in the infarcted area (1 day after MI) was reduced in both the Met-CCL5-FDH (272 ± 35.8 per mm^2^) and the Met-CCL5-FDH+CXCL12 (S4V)-SDH groups (299 ± 33.6 per mm^2^), but not in the CXCL12 (S4V)-SDH group (876 ± 235.9 per mm^2^), compared with the control groups that received only hydrogel (810 ± 51.3 per mm^2^) or PBS (760.7 ± 72.2 per mm^2^; Fig. 3A and B). Similar results were obtained by staining with anti-MPO antibody (Fig. 3C and D), confirming the specificity of the administration of the CCR1 and CCR5 antagonist Met-CCL5 for the prevention of neutrophil recruitment.

Similarly, neovascularization 4 weeks after MI was also improved in both the CXCL12 (S4V)-SDH (788 ± 28.8 per mm^2^) and the Met-CCL5-FDH+CXCL12 (S4V)-SDH groups (1022 ± 63.1 per mm^2^), to a higher extent as the Met-CCL5-FDH (466 ± 29.4 per mm^2^), hydrogel group (328 ± 32.5 per mm^2^) and control groups (407 ± 62.4 per mm^2^), as quantified by CD31-staining (Fig. 4A and B). This indicates a CXCL12 (S4V)-mediated increase in angiogenesis in infarcted myocardium, supporting our hypothesis. To further investigate the pro-angiogenic signalling, we performed immunohistological staining for the downstream of CXCL12-induced activation of Akt (through phosphorylated-Akt) and we observed a higher activation after CXCL12 (S4V) and Met-CCL5-FDH+CXCL12 (S4V), 1 day after MI (Fig. 4C) sustaining even up to 4 weeks after MI (Fig. 4D), compared with control and Met-CCL5–treated groups. These findings suggest that the effects mediated by the CXCL12/CXCR4 pathway, *e.g*. the recruitment of hematopoietic cells and the activation of angiogenic pathways, would serve to reduce infarct size in our study rather than the blockade of initial neutrophil infiltration.

**Fig. 5:**
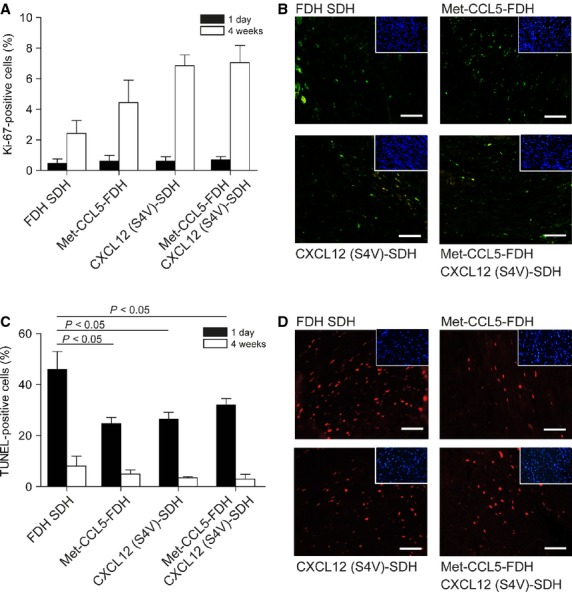
Assessment of apoptosis and proliferation after experimental myocardial infarction (MI). Proliferating cells were stained with Ki67 and quantified as per cent from total cells, 1 day (black bars) and 4 weeks (white bars) after MI (**A**). Representative pictures of positive Ki67 staining 4 weeks after MI are showed (**B**, DAPI staining in insets). Apoptotic cells were stained and quantified as per cent of total cells, 1 day (black bars) or 4 weeks (white bars) after MI (**C**). Representative pictures of positive TUNEL staining 1 day after MI are shown (**D**, DAPI staining in insets); scale bars: 50 μm. Depicted P values are based on parametric anova (*n* ≥ 6).

Finally, we did not observe significant differences in (endothelial cell and cardiomyocyte) proliferation, 1 day and 4 weeks after MI, despite a trend towards increased Ki67-staining in the CXCL12 (S4V) and Met-CCL5-FDH+CXCL12 (S4V) groups compared with the control group (Fig. 5A and B). However, all chemokine-treated groups showed less apoptotic cells than the control at the end-points of the study (Fig. 5C and D) suggesting an accelerated wound healing in these groups.

## Discussion

Chemokines modulate all phases of MI, having a decisive role in ventricular remodelling, healing and scar formation [1,2]. Some of them with cardioprotective functions (*e.g*. CCL2, CXCL12, Macrophage Migration Inhibitory Factor) are rapidly up-regulated to rescue the cardiomyocytes from the imminent ischemic injury. Other chemokines initiate an inflammatory reaction, recruiting neutrophils (CCR1-ligands) or inflammatory monocytes (CCR2-ligands) that clean the injured area from cellular debris. Later, chemokines, *e.g*. CX3CL1 or CCR5-ligands, promote healing by stimulating collagen deposition and angiogenesis [1,2].

In this context, we have proposed a novel strategy of combining the Met-CCL5 and protease-resistant CXCL12 treatments, for the simultaneous activation of two important mechanisms for preservation of heart function: inhibition of neutrophil infiltration and enhanced neovascularization by increasing the recruitment of hematopoietic stem cells. Met-CCL5 antagonizes CCR1 and CCR5 activation and function in response to their natural ligands CCL3-5, and this blockade is able to reduce inflammation in models of induced inflammatory and autoimmune diseases, but also after MI [8]. Recombinant Met-CCL5 chemokine was formulated in a synthetic, biodegradable hydrogel for a fast release and was shown to reduce inflammation and the migration of neutrophils during the first hours after MI. Combined treatment with CXCL12 (S4V) in a slowly degrading gel improved the heart function, decreased infarction area and increased capillary density 4 weeks after MI compared with control group.

Currently, the majority of small and large molecular drugs are delivered into patients systemically (*e.g*. oral or intravenous release) without the use of a scaffold. Consequently, large doses are usually required for a desired local effect because of non-specific uptake of other tissue, which can lead to serious side effects. Thus, biodegradable hydrogels are utilized here to stabilize and deliver bioactive molecules in the desired tissue for a better dose monitoring and an exact release [14] allowing a local and specific release of Met-CCL5 and protease-resistant CXCL12 into the heart tissue over 24 hrs or 4 weeks respectively. Natural CXCL12 was broadly used in models of MI. However, we found that the injection of CXCL12 after MI has only limited results [24]. One reason for the inefficiency of natural CXCL12 might be the rapid cleavage by MMP-2, resulting in a tetrapeptide and a neurotoxic CXCL12 remnant [25]. Until now, the physiological role of this cleavage is unknown, yet CXCL12 inactivation by MMPs stops mobilization of hematopoietic stem cells from the bone marrow [13]. Up-regulation of MMP-2 after MI is well known and plays an important role in the extracellular matrix turnover and remodelling [26]. Therefore, to sustain the CXCL12 function *in vivo,* it is essential to block its degradation. For our herein described mouse *in vivo* study, we implemented a previously described protease-resistant CXCL12 variant to investigate the improvement of cardiac function after MI [13]. When incorporated into a slowly degradable hydrogel, CXCL12 is released gradually over a longer time period, assuring a local high amount of chemokine and a continuous recruitment of stem cells. Accordingly, neo-angiogenesis after MI was increased after treatment with CXCL12 (S4V)-containing hydrogels, demonstrating the efficiency of the treatment. Although CXCL12 is also involved in retaining neutrophils in the bone marrow [27], the exogenous addition of CXCL12 would increase the amount of circulating neutrophils. However, as the biodegradable hydrogel warrants a local and controlled action of CXCL12, this pro-inflammatory function of CXCL12 is expected to be minor in our experimental setting.

On the other hand, to reduce inflammation induced by MI, recombinant Met-CCL5 was used to inhibit neutrophil infiltration. It is well-established that CXCL8 and CCL5 are strong agonists for neutrophil recruitment. Met-CCL5 is able to antagonize the actions of CCL5 not only *in vitro* but also *in vivo* [8]. As the infiltration of neutrophils occurs only for a short time period [23], we combined Met-CCL5 with a very quickly biodegradable hydrogel, which assures the release of this antagonist only for several hours. This is essential to avoid the blocking of later functions of CCL5, *e.g*. over CCR5, such as recruitment of reparatory monocytes or T regulatory cells [28], which is necessary for a proper healing and scar formation.

By the combined treatment with both recombinant chemokines formulated in a time-dependent degradable hydrogel, we significantly preserved the heart function and improved remodelling of the ventricle. The cardioprotective effects of the Met-CCL5 and protease-resistant CXCL12 are mediated through reduced infiltration of neutrophils in the infarcted myocardium, reduced apoptosis and increased recruitment of hematopoietic stem cells respectively. Indeed, we found a decreased neutrophil infiltration after treatment with Met-CCL5-FDH, and after combined treatment with Met-CCL5-FDH and protease-resistant CXCL12 (S4V)-SDH. These results are comparable with those found in CCR1-deficient mice, or after anti-CCL5 mAb treatment, which showed that the initial inhibition of CCL5 during early MI is cardioprotective owing to its anti-inflammatory effects [8,9], reducing both infarct size and post-infarction heart failure in mouse model of chronic cardiac ischaemia [29].

Moreover, increased angiogenesis was noticed after CXCL12 (S4V)-SDH alone and combined CXCL12 (S4V)-SDH and Met-CCL5-FDH treatment, suggesting that the preserved ability of protease-resistant CXCL12 mediated stem cell recruitment through its CXCR4 receptor, which might be demonstrated by the increased phosho-Akt staining in the CXCL12-treated groups. As expected, Met-CCL5 alone was not able to promote angiogenesis. However, although a decrease in infarction size was observed in all treated groups, it appears that only combined treatment with CXCL12 (S4V) and Met-CCL5 is able to additionally improve the heart function and to assure the best healing and remodelling of the ventricle after MI.

## Conclusions

In summary, our study provides evidence that the combined therapy of the protease-resistant CXCL12-SDH and Met-CCL5-FDH preserves cardiac function, promotes angiogenesis and facilitates wound healing processes by attenuating neutrophil-induced myocardial inflammation, representing an advantage over established separate therapies implementing CXCL12 and Met-CCL5. This novel strategy might constitute an additional option to optimize cardiac repair and remodelling after myocardial injury and might complement cell-based therapies.

## Supporting information

**Figure 1** Synthesis of biodegradable hydrogels. **Figure 2** Met-CCL5 isolation, identity and mass determination. **Figure 3** CXCL12 (S4V and S2G4V) isolation, identity and mass determination. **Figure 4** Influence of chemokines and biopolymer on cell viability. **Figure 5** Concentrations of chemokines in mouse sera.
