# Supplementary material for: Controlled intramyocardial release of engineered chemokines by biodegradable hydrogels as a treatment approach of myocardial infarction
Source: J Cell Mol Med. 2014 Feb 6;18(5):790–800. doi: 10.1111/jcmm.12225 (PMC4119385; doi:10.1111/jcmm.12225)
Supplement: Supplementary file 1 [file jcmm0018-0790-SD1.doc]

# Controlled intramyocardial release of engineered chemokines by biodegradable hydrogels as a treatment approach of myocardial infarction

# Delia Projahn, Sakine Simsekyilmaz, Smriti Singh, Isabella Kanzler, Birgit K. Kramp, Marcella Langer, Alexandrina Burlacu, Jürgen Bernhagen, Doris Klee, Alma Zernecke, Tilman M. Hackeng, Jürgen Groll, Christian Weber, Elisa A. Liehn, Rory R. Koenen

**Supplementary Figures**


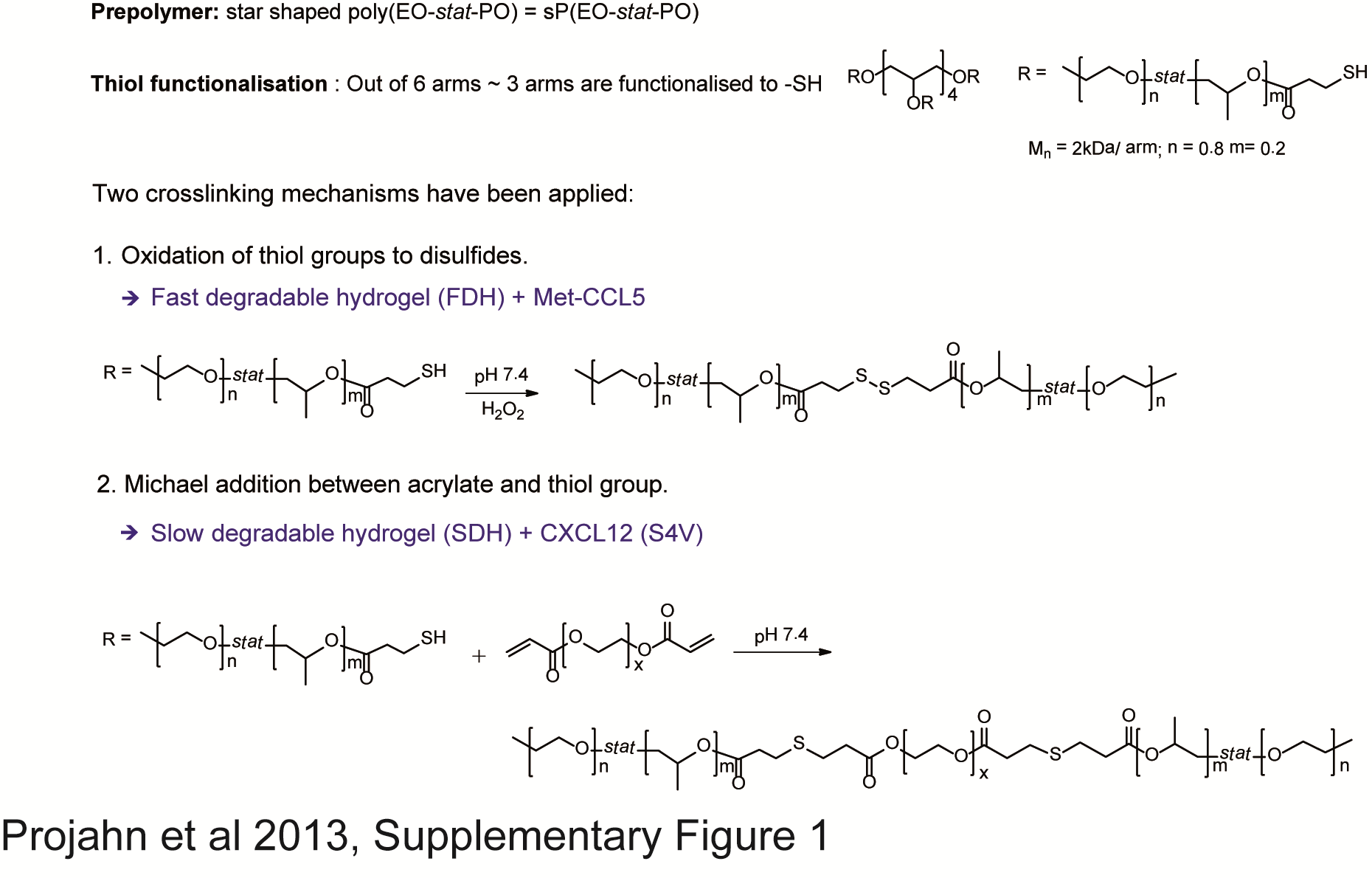


**Supplementary Figure 1:** *Synthesis of biodegradable hydrogels.*

The thiolated polymer termed sP(EO-*stat*-PO) was crosslinked by two mechanisms: The first mechanism is an oxidation (by H_2_O_2_) of thiol groups to disulfides, which represents the FDH and is mixed with Met-CCL5. The second mechanism is a Michael addition between acrylate and thiol group, which represents the SDH and is mixed with CXCL12.


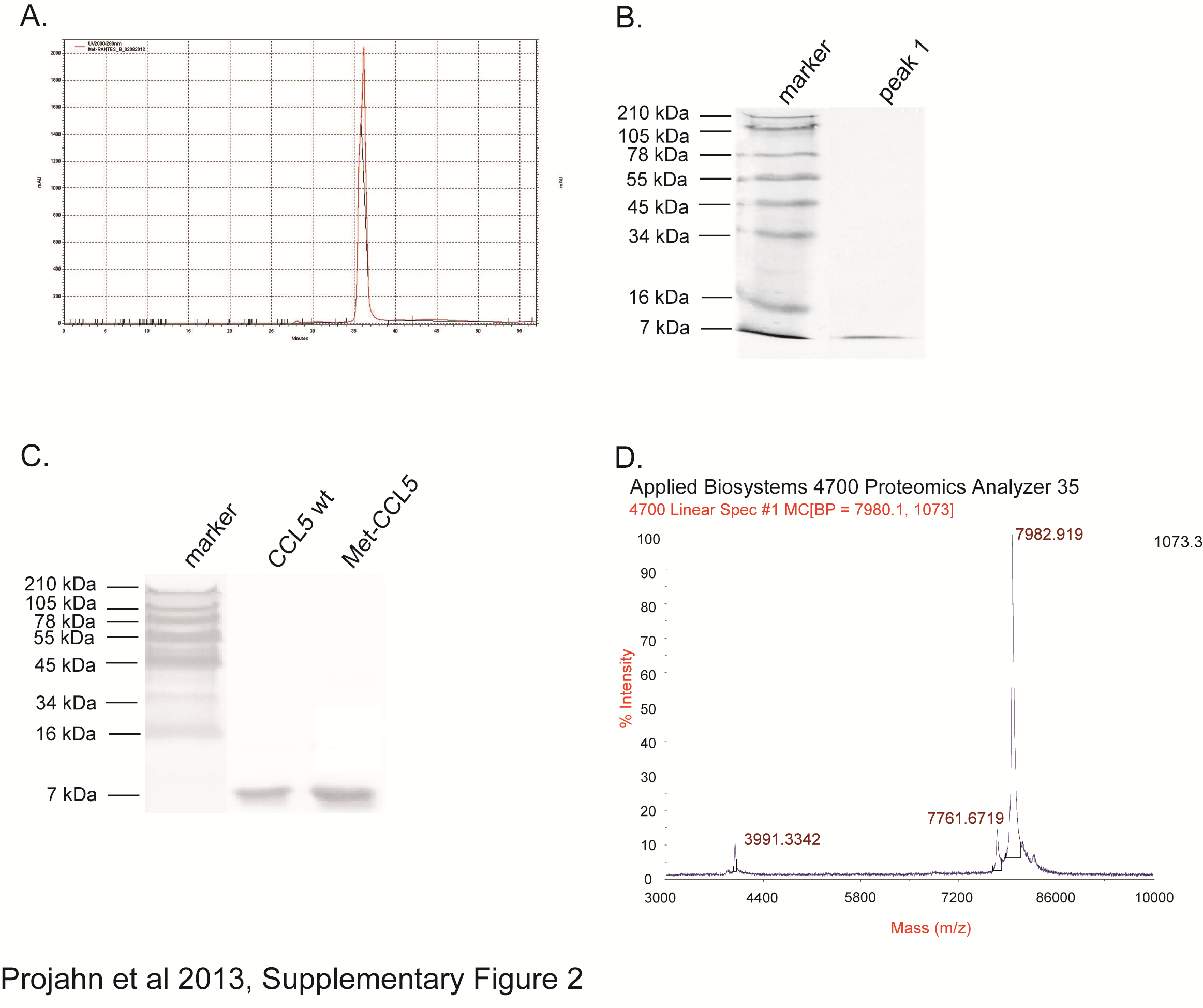


**Supplementary Figure 2:** *Met-CCL5 isolation, identity and mass determination.*

**A.** Representative HPLC trace of the last purification step for Met-CCL5. A single symmetric peak indicates purity of the material. **B.** Coomassie staining for Met-CCL5 chemokine. SDS-PAGE was performed under reducing conditions. **C.** Western blot analysis of the isolated Met-CCL5 chemokine compared with the commercial available CCL5 *wild-type*. **D.** Purity and mass determination of Met-CCL5 chemokine by means of MALDI-TOF. The mass of Met-CCL5 chemokine corresponds to 7.98 kDa.


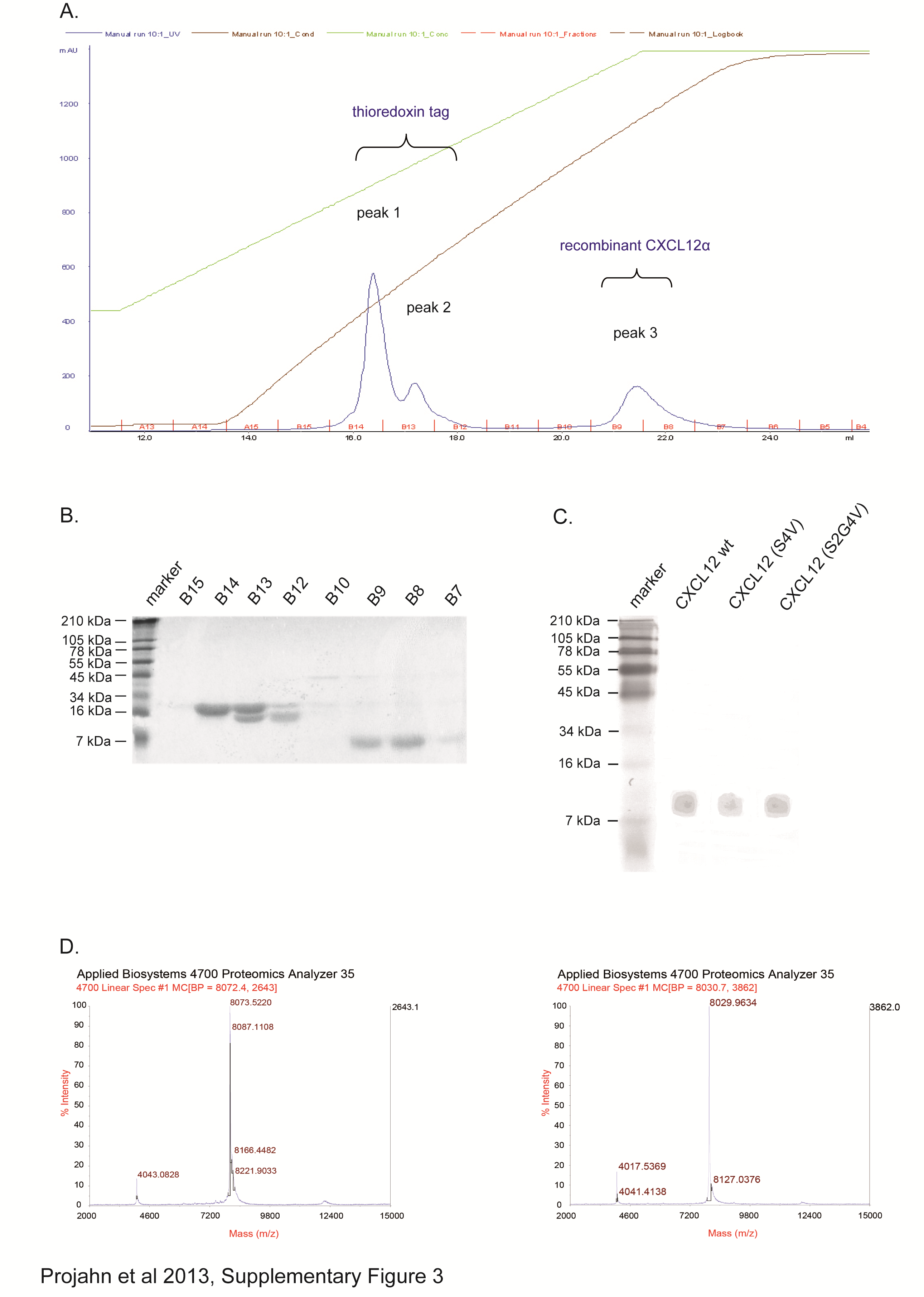


**Supplementary Figure 3:** *CXCL12 (S4V and S2G4V) isolation, identity and mass determination.*

**A.** Representative cation exchange chromatography trace of the last purification step for CXCL12 (S4V). Peak 3 represents the recombinant CXCL12 (S4V). Peak 1 and 2 are cleavage products. **B.** Coomassie staining for CXCL12 (S4V) chemokine. SDS-PAGE was performed under reducing conditions. **C.** Western blot analysis of the isolated CXCL12 (S4V and S2G4V) chemokines compared with the commercial available CXCL12 *wild-type*. **D.** Purity and mass determination of CXCL12 (S4V and S2G4V) chemokines by means of MALDI-TOF. **D.** The mass of CXCL12 (S4V) corresponds to 8.07 kDa and CXCL12 (S2G4V) corresponds to 8.03 kDa.


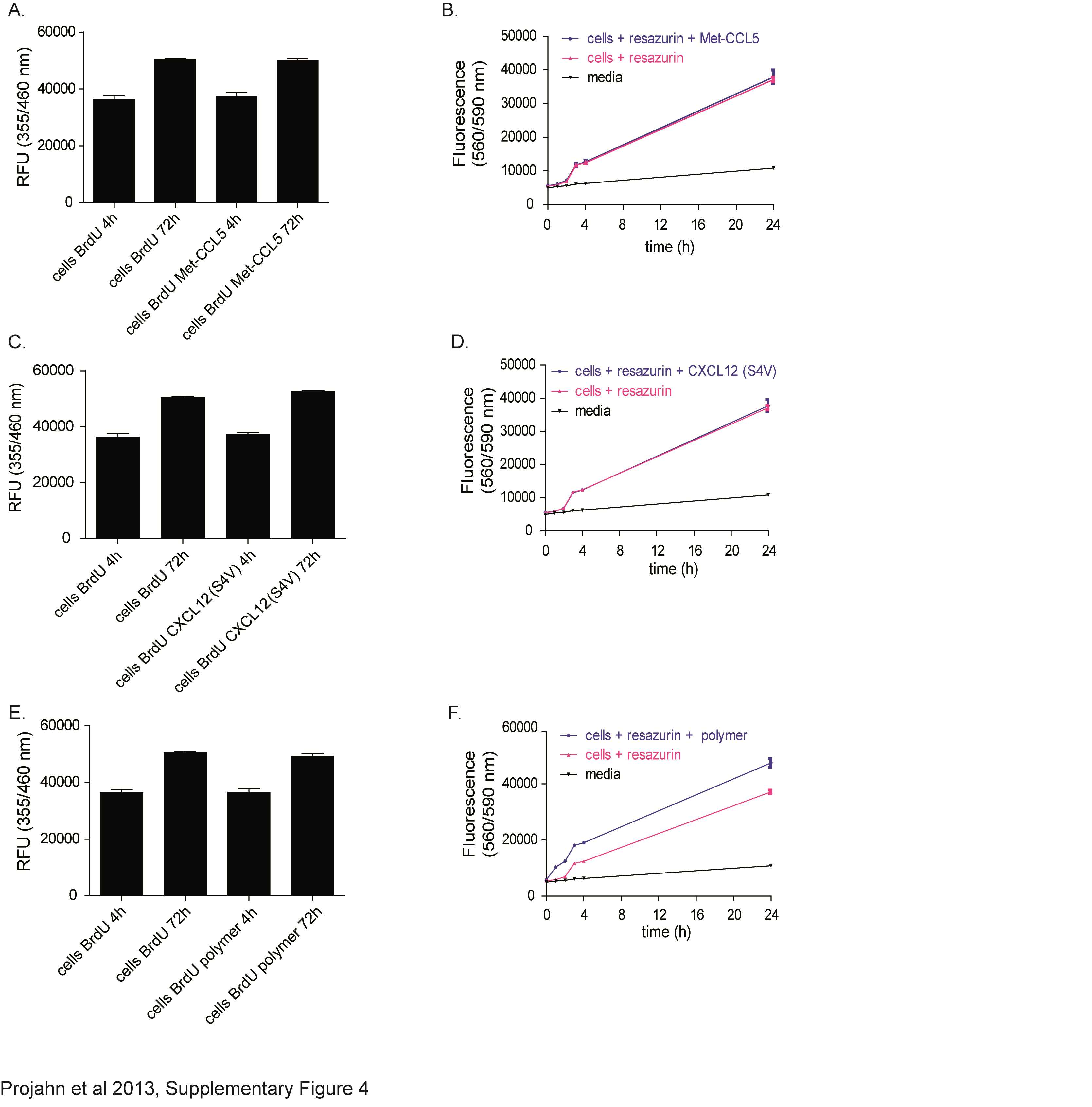


**Supplementary Figure 4:** *Influence of chemokines and biopolymer on cell viability.*

Proliferation assay **(A,C,E)** and cell viability assay **(B,D,F)** of BrdU labeled HUVEC (1000 cells/well) incubated with/without Met-CCL5 **(A,B)**, CXCL12 (S4V) **(C,D)** or degradable polymer **(E,F)** until 24 or 72 h.


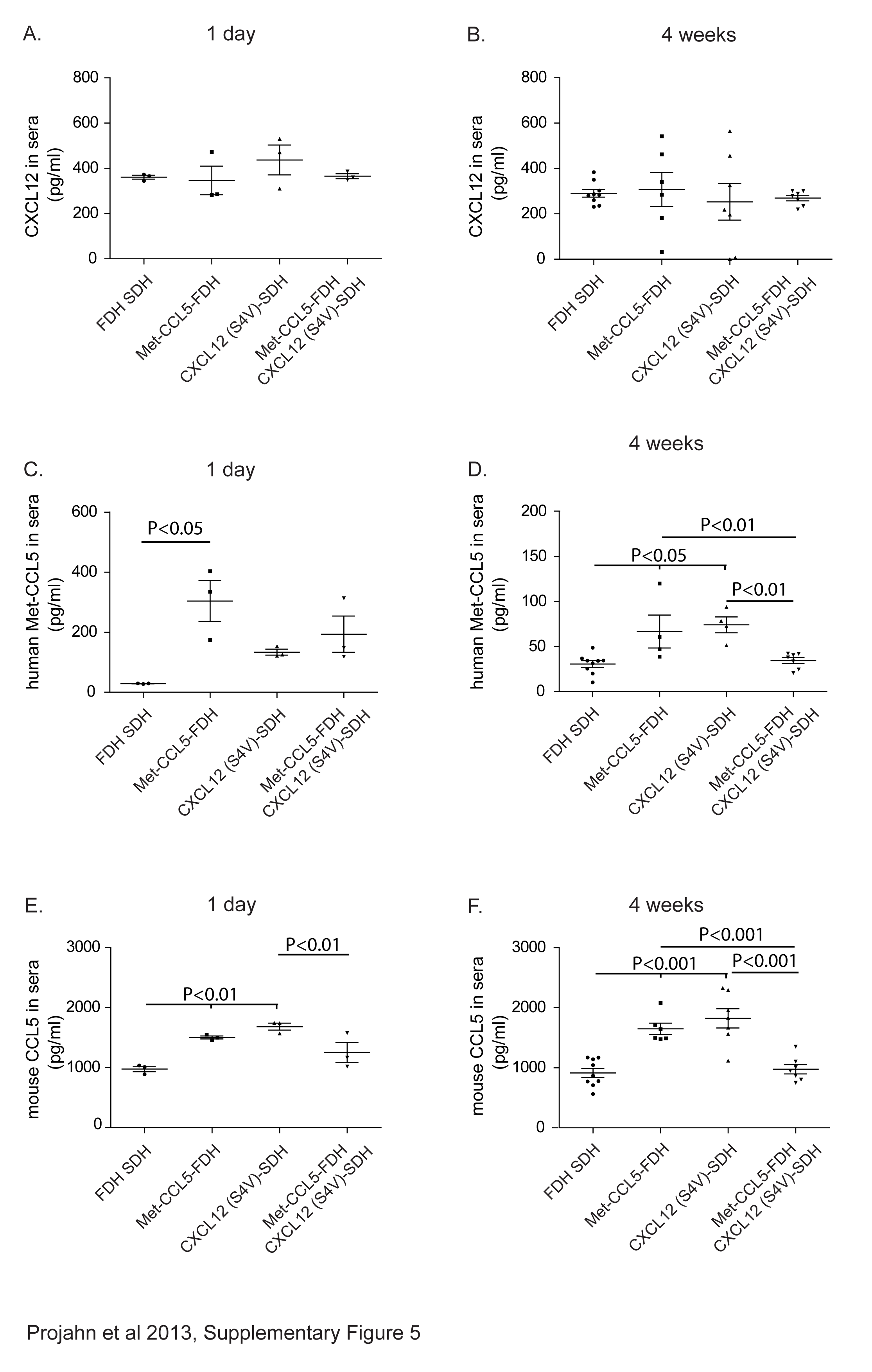


**Supplementary Figure 5:** *Concentrations of chemokines in mouse sera.*

Detection of the amount of mouse CXCL12, human Met-CCL5 and mouse Met-CCL5 in mouse sera using ELISA. Depicted P-values are based on ANOVA (n=6-9).
